# Measuring Gaze and Arrow Cuing Effects With a Short Test Adapted to Brain Damaged Patients With Unilateral Spatial Neglect: A Preliminary Study

**DOI:** 10.3389/fpsyg.2021.690197

**Published:** 2021-07-29

**Authors:** Rindra Narison, Marie de Montalembert, Andrew Bayliss, Laurence Conty

**Affiliations:** ^1^Laboratory of Cognitive Functioning and Dysfunctioning (DysCo), Univ. Paris Nanterre, Nanterre, France; ^2^Rehabilitation Center of “Le Bourbonnais” and SAMSAH UGECAM BFC, Bourbon Lancy, France; ^3^School of Psychology, University of East Anglia, Norwich Research Park, Norwich, United Kingdom

**Keywords:** gaze cuing, arrow cuing, gaze liking effect, right brain lesion, left unilateral spatial neglect

## Abstract

People with left unilateral spatial neglect (USN) following a right brain lesion show difficulty in orienting their attention toward stimuli presented on the left. However, cuing the stimuli with gaze direction or a pointing arrow can help some of them to compensate for this difficulty. In order to build a tool that helps to identify these patients, we needed a short version of the paradigm classically used to test gaze and arow cuing effects in healthy adults, adapted to the capacities of patients with severe attention deficit. Here, we tested the robustness of the cuing effects measured by such a short version in 48 young adult healthy participants, 46 older healthy participants, 10 patients with left USN following a right brain lesion (USN+), and 10 patients with right brain lesions but no USN (USN–). We observed gaze and arrow cuing effects in all populations, independently of age and presence or absence of a right brain lesion. In the neglect field, the USN+ group showed event greater cuing effect than older healthy participants and the USN– group. We showed that gaze and arrow cuing effects are powerful enough to be detected in a very short test adapted to the capacities of older patients with severe attention deficits, which increases their applicability in rehabilitation settings. We further concluded that our test is a suitable basis to develop a tool that will help neuropsychologists to identify USN patients who respond to gaze and/or arrow cuing in their neglect field.

## Introduction

Unilateral spatial neglect (USN) involves a difficulty to detect, respond to and orient one's attention toward stimuli presented to (or represented on) the contralateral side of a brain lesion, which is usually located in the right hemisphere (Heilman et al., 1993). As USN hampers individuals' ability to recover their autonomy, several rehabilitation techniques have been proposed to reduce USN (e.g., Luauté et al., 2006). However, in a Cochrane review, Bowen et al. (2013) highlighted the limited effect of these techniques for daily activities and the need to rely on patients' preserved abilities during rehabilitation. When the brain is undamaged, adults spontaneously follow others' gaze direction and arrows toward the surrounding space (for a review, Frischen et al., 2007). The resulting cuing effects play an important role in normal cognition, especially those related to others' gaze (Csibra and Gergely, 2009). Few researches have investigated gaze and arrow cuing effects in patients with USN, and those studies reported inconsistent results (see Vuilleumier, 2002, Bonato et al., 2009). Recently, we defended the view that the high heterogeneity of the lesions causing USN (Chechlacz et al., 2012; Molenberghs et al., 2012) predicts a high heterogeneity in the preservation of gaze and arrow cuing effects, which are subtended, at least partly, by specific brain mechanisms (e.g., Lockhofen et al., 2014; Sato et al., 2016; Zhao et al., 2017). We thus started to develop a method to identify USN patients who respond to gaze and/or arrows in their neglect field.

Our purpose is not to develop a new procedure to diagnose spatial neglect, but to identify the patients with USN who may benefit from cuing effects as a base for compensation during rehabilitation (Narison et al., 2019). In the case a patient is identified as a gaze responder, the patrician, family and/or caregiver would know that they can use their gaze efficiently during interactions to stimulate the patient in exploring his/her neglected field. In the case where the patient is identified as an arrow responder, the patrician can recommend the family to hang left arrow on the wall of the patient's bedroom, to signal to the patient the presence of significant elements. Future trainings should also be created to reeducate cuing effects in non-responder patients. However, developing a functional pronostic tool in patients with USN requires a short, simple version of the paradigm classically used to measure gaze and arrow cuing effects in adults (i.e., the Posner-like paradigm; Posner, 1980), since several tests are already administered to patients with USN who show high fatigability. Here, we tested whether such a brief version allows to measure robust cuing effects.

In the Posner-like paradigm, the participant's task is either to detect, discriminate or categorize a target appearing on a computer screen by pressing the correct response key as quickly as possible. The target appearance is preceded by a central cue indicating right or left. Then, the target appears either on the side indicated by the central cue (congruent condition) or on the opposite side (incongruent condition). Typically, the central cue may be a face looking straight ahead with eyes deviating to one side, or a horizontal bar evolving into an arrow. Sometimes, authors also manipulate a neutral condition in which the central cue does not indicate the left or the right (e.g., a face looking straight ahead or squinting). Not surprisingly, previous studies showed that congruent trials are processed faster than incongruent or neutral ones, independent of task type (for a review, see Frischen et al., 2007). Using at least 20 trials per experimental condition (e.g., Bayliss et al., 2010), but usually many more (e.g., McCrackin and Itier, 2019), these effects were largely reproduced in healthy adults. Here, we question the applicability of gaze and arrow cuing effects measured with the Posner like paradigm in a patient population, specifically patients with USN.

In Narison et al. (2019), we investigated gaze and arrow cuing using Congruent, Incongruent and Neutral conditions. We demonstrated that contrasting Congruent to Neutral conditions led to higher cuing effects than contrasting Congruent to Incongruent conditions in healthy adults. We also showed that incongruent cues yielded issue of attention disengagement among patients with right brain damage (see also Bartolomeo et al., 2001; Dalmaso et al., 2015, for a review, see Bartolomeo and Chokron, 2002). Thus, here, we decided to use only congruent and neutral conditions to reduce time testing. We also determined that the test should be administered twice (i.e., in 2 independent sessions) to avoid false positive (i.e., stating by error that a patient respond to gaze and/or arrow cuing in the neglect side). This requires us to limit the number of trials per session. Given the need of two sessions and the fatigability of the target participants, we decided to use only 10 trials per condition and tested whether cuing effects can be measured in this context, which would increase their applicability in rehabilitation settings.

We previously demonstrated that gaze and arrow cuing effect follow a Gaussian distribution among healthy people. Such distribution is useful in neuropsychology, as it can serve as a reference to identify when patients' performance deviates (or not) from the norm (Amieva et al., 2011). At term, to calculate this norm, we might have to calibrate the tool that we aim at developing in a wide range of ages among healthy people. Indeed, USN may occur at any age (Gottesman et al., 2008) and cuing effects could evolve with aging. Some studies have investigated cuing effects in older people and argue that a specific age-related decline occurs for gaze cuing (e.g., Slessor et al., 2008; Bailey et al., 2014). By contrast, other authors argue that cuing effects do not decline with aging as long as the time manipulated between the cue and the target appearance was adapted to executive abilities of older participants (i.e. cue target onset asynchrony or CTOA > 300 ms). Therefore, we tested our short version of the Posner-like paradigm in two control groups, young and older healthy adults. We chose a CTOA of 500 ms and tested whether the cuing effects measured by our test was robust in both groups and/or declined with aging.

Beyond testing our short test in healthy control participants, we also tested it in patients with right brain damage and a diagnosis of USN to ensure that the test was adapted to this target population. As a supplementary control group, and to disentangle effects related to USN from effects related to right brain damage, we also tested patients with right brain damage but no USN, a population that has previously been reported to respond to gaze and arrow cuing (Bonato et al., 2009; Dalmaso et al., 2015). Based on Narison et al. (2019), we expected mean gaze and arrow cuing effects to be detectable in all groups of participants. We also expected that most patients with USN would spontaneously use central cues to compensate for their neglect. Thus, on average, we should observe greater cuing effects in their neglect field when compared to their right field and to participants without USN.

## Methods

### Participants

A total of 114 right-handed native French-speaking participants were included in the study: 10 patients diagnosed with left USN (USN+) secondary to right brain damage, 10 patients with right brain damage but no left USN, 46 healthy older participants and 48 healthy young participants (see Table 1). Patients (with and without left USN) were recruited from the neurological rehabilitation unit of “Centre de rééducation et de réadaptation fonctionnelles Le Bourbonnais UGECAM BFC” at Bourbon Lancy (France, 71). A full description of the patient group is presented in Table 2. A neuropsychologist and a physician both specialized in USN have assigned the diagnosis of left USN to patients, based on clinical observation, lesion localization, and behavioral and neuropsychological tests (see Table 2 for full details about the neuropsychological tests). Patients were excluded if they were judged to be unable to understand task instructions, if they had multiple brain lesions, or a history of psychological or psychiatric disorders.

**Table 1:** Gender distribution (F for Females, M for Males) and age (mean ± standard error) for each group.

	**Gender**	**Age**	**Age difference between patients and older controls**
Young Controls	26F/22M	24.5 ± 0.8	
Older Controls	24F/22M	62.8 ± 1.5	*F*_(2, 12.8)_ = 1.28, *p* > 0.3
USN+ Group	5F/5M	68.3 ± 3.0	
USN– Group	4F/6M	55.6 ± 6.6	

*Note that the variances of the variable Age were not homogeneous between groups (Levene's test, p < 0.001). The right column shows the result of the Welch's ANOVA run on the variable Age with Group as between subject factors (Older Controls, USN– Group, USN+ Group), revealing that groups did not differ on this variable. Moreover, importantly, none of the reported results were modulated by the age of the participants, when introducing this variable as a regressor in the statistical model (ANCOVA). This showed that the difference of variance in age between USN+ group and its control groups (USN– Group and Older Controls) cannot explain the differences observed between those groups*.

**Table 2:** Characteristics of patients with right brain damage.

**Case *n*^**°**^**	**Group patient**	**Sex**	**Age in years**	**Laterality**	**Etiology**	**Lesion**	**Delay (in months)**	**LHH**	**L-R bell's omissions**	**First bell column**	**Line bisection deviation (mm)**	**Visual field Tap L-R omissions**	**Neglect TAP L-R omissions**
2	USN+	F	67	Amb	lob	Fronto-sub cortical	3	A	14*	7*	−4	0	7*
3	USN+	F	62	R	hem	Frontal	1.5	A	0	4*	−0.5	0	2
4	USN+	F	71	Amb	hem	Lenticular nucleus	36	P	4*	2	12.5*	0	8*
7	USN+	F	77	R	isch	Lateral sulcus	3	P	6*	7*	40*	6*	5*
12	USN+	M	57	R	isch	Parieto-sub cortical	1	A	6*	7*	10.5*	3*	9*
15	USN+	M	50	R	isch	Lateral sulcus	1.5	P	5*	7*	30.5*	12*	2
19	USN+	M	80	R	isch	Internal capsule	5	P	2*	7*	2	8*	8*
20	USN+	M	74	R	isch	Lateral sulcus	7.5	P	5*	7*	−3.5	1*	4*
18	USN+	F	68	R	isch	Fronto-parietal	1	A	6*	7*	0	0	10*
21	USN+	M	67	R	hem	Fronto-sub cortical	2	P	8*	7*	27.5*	2*	10*
5	USN–	M	37	R	hem	Capsulo-lenticular	13	A	−1	1	−3.5	0	0
6	USN–	F	39	R	tum	Frontal	72	A	6*	1	3.5	−1*	0
8	USN–	M	67	R	hem	Fronto-parietal	3	A	4*	2	−2	−2*	8*
9	USN–	M	79	Amb	hem	Lenticulo-capsulo-thalamic	2	A	0	2	0.5	0	0
10	USN–	F	71	R	isch	Caudate + Lenticular nucleus	1	A	−2*	2	−3	0	−2
11	USN–	F	27	R	absc	Parietal	3.5	A	0	2	−6	0	−2
13	USN–	M	30	R	isch	Lateral sulcus	1	A	−1	1	−5	0	−1
14	USN–	M	84	R	isch	Fronto-parieto-occipital	1.5	A	2*	7*	13*	0	0
16	USN–	M	64	R	isch	Posterior lateral sulcus	3	A	−1	5*	3	0	−1
17	USN–	F	58	R	hem	Frontal	8	A	0	7*	0	−2*	0

The presence (USN+) or absence (USN–) of neglect symptoms was determined based on clinical observation, lesion localization, and behavioral and neuropsychological tests. Patients underwent a neuropsychological evaluation testing episodic memory with the RL/RI-16 items (Van der Linden et al., 2004), executive functions with the Grefex Battery (Godefroy, 2008), attentional functions with the TAP (Zimmermann and Fimm, 2010) and instrumental functions with the VOSP Test, visuo-constructive reproduction and DO80 (Deloche and Hannequin, 1997). Neglect symptoms were tested with the Bell Test, the line bisection and the Ogden scene from “Batterie d'Evaluation de la Négligence Unilatérale du Geren” (BEN, Azouvi et al, 2002). Moreover, we used two subtests from the TAP (Zimmermann and Fimm, 2010) to further support the diagnosis: visual field examination and examination of visual field “neglect condition.” Sex (F = female, M = male), Laterality (R = right-handed, Amb = ambidextrous), Etiology (TBI = traumatic brain injury, Isch = ischemia, Hem = hemorrhage, lob = lobectomy), LHH: left homonymous hemianopia (A = absent, P = present, NE: not analyzable), L-R bell's omissions: difference in omissions between the left and right fields on Bell's Test (positive value = more omissions on the left, negative value = more omissions on the right), First bell column: Column of the first found bell, Line Bisection deviation in millimeters (positive value = deviation toward the right, negative value = deviation toward the left), Visual Field TAP L-R omissions: difference in omissions between the left and right fields on the TAP “Visual Field” sub-test (positive value = more omissions on the left, negative value = more omissions on the right), Neglect TAP L-R omissions: difference in omissions between the left and right fields on the TAP “Neglect” sub-test (positive value = more omissions on the left, negative value = more omissions on the right), Pathological scores on neuropsychological tests are indicated with ^*^. The delay between the stroke onset and the current test is indicated in months.

All participants were naive to the aim of the experiment and had normal or corrected-to-normal visual acuity. All participants provided written informed consent according to institutional ethics committee guidelines, and in compliance with the Declaration of Helsinki. The procedure was approved by the local ethics committee (CPP Est I, approval no. 2016-A01433-48). Healthy participants had no neurologic or psychiatric history. To be included, healthy older participants needed to score below 5 on the 15-item Geriatric Depression Scale (Clément et al., 1997) and above the 5th percentile on the Mini Mental State Examination (Kalafat et al., 2003).

### Stimuli

#### Gaze Cues

Face stimuli consisted of 20 static color photographs of 10 individuals (5 males/5 females) selected from a database of digitized portraits of adult faces (see Conty et al., 2007). All faces were of individuals unknown to our participants and had a neutral expression. Head direction was always oriented straight toward the observer. Each individual was presented in two different views: one with the eyes directed straight toward the observer (Direct Gaze), and one with the eyes averted 30° toward the right side of the observer's position (Averted Gaze). To avoid any unintended differences in picture backgrounds, the eye region in the averted gaze stimuli was cut and pasted into the very same position within the photographs used for the direct gaze stimuli. Left sides for all stimuli were obtained by mirror-imaging. All stimuli were presented in 256 colors and reduced to a height of 310 pixels and a width of 148 pixels while preserving their proportion. During the experiment, the face stimuli covered a visual angle of approximatively 7.5° vertically and 6° horizontally.

#### Arrow Cues

Arrows were created using Photoshop CS5.1. Three pictures were created. The first picture represented a white bar measuring 112 pixels (width) x 12 pixels (height) superimposed on a white circle (Ø 56 pixels). The second and third pictures were the same but with an arrow pointing toward the right or left instead of the bar. These objects were designed to cover the eye region of the faces, i.e., approximately a visual angle of 1.5° vertically and 3° horizontally.

#### Target

The target stimuli consisted of 12 pictures of kitchen utensils selected from a database of household objects (Bayliss et al., 2006). Each object was available in four colors (green, yellow, red, blue), and we chose the blue ones. While preserving their proportions, the pictures were resized to cover between 1° and 4.5° of visual angle horizontally and between 3.5° and 5.5° of visual angle vertically during the experiment. The 12 objects were split into 6 pairs of 2 objects, one large with a handle and one small. The 6 pairs were the following: coffee maker/ladle; thermos flask/pizza wheel; kettle/ice cream spoon; iron/tea strainer; shaker/spoon; teapot/spatula). During the experiment, the objects were always presented vertically with handle (when applicable) oriented to the left. Indeed, Di Pellegrino et al. (2005) demonstrated that objects with handles affording a left-hand grasp reduce USN.

### Procedure

Participants sat approximately 60 cm from a 15.6-inch computer screen (with a resolution of 1366 × 768 pixels) on which stimuli were shown on a black background. E-Prime® 2.0 software was used to control stimulus presentation, response recording and latency. The screen height was adjusted so that the middle of the screen was aligned with participants' eyes. The experiment was divided into three parts. Here, we presented the main (first) test. Two supplementary short tests are presented in Supplementary Material.

During the main test, participants completed 84 trials of the classical Posner-like paradigm aimed at investigating gaze and arrow cuing effects (mean test duration: 7 min). On each trial, participants had to indicate as fast and correctly as possible whether an object (the target) appeared on the left or on the right of a computer screen by pressing one of the two corresponding mouse buttons. A cue always preceded the object's appearance. We used 3 cue conditions which were either congruent (i.e., indicating the side of the target's appearance) or neutral: The Gaze condition (20 congruent trials: 10 with left averted gaze, 10 with right averted gaze), Arrow condition (20 congruent trials: 10 with left pointing arrow, 10 with right pointing arrow) and Neutral condition (20 trials: 10 with the target appearing on the left, 10 with the target appearing on the right). Since we manipulated only congruent cues, the cue predicted the side of the target appearance. In order to avoid anticipated responses, we added 24 Catch trials (8 in each of the 3 cue conditions) in which no target appeared. Participants were instructed not to respond to those trials. As trial presentation was randomized across participants, catch trials required that participants wait for the target's appearance before providing a response.

**Figure 1:**
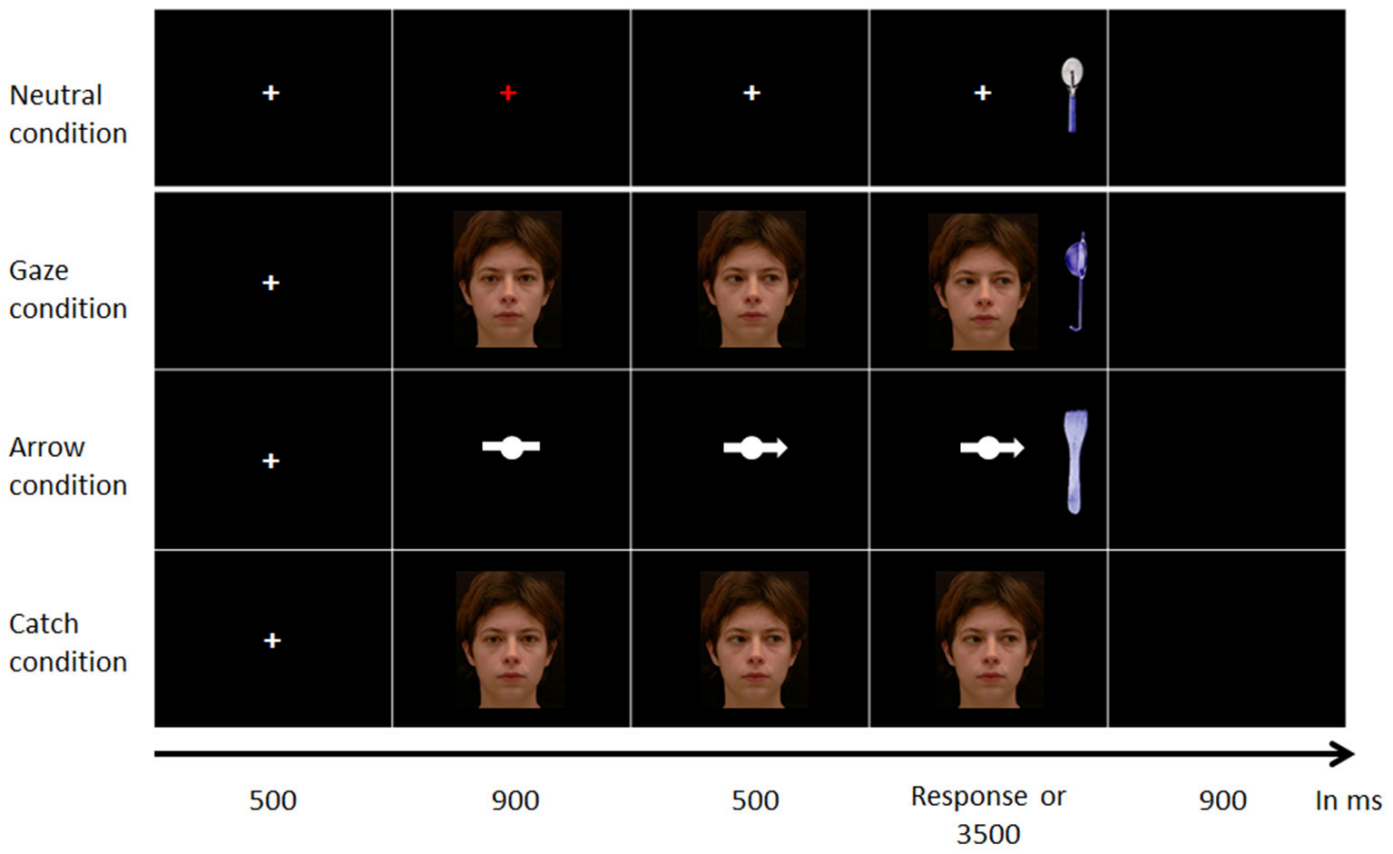
Illustration of the time course of the experimental trials in part 1. Time course for one trial of the Neutral condition (upper line), one trial of the Gaze condition (second line), one trial of the Arrow condition (third line) and one catch trial (bottom line). Participants were asked to maintain their attention on the screen's center until the object appeared, at which time they were free to initiate eye movements. The experimenter was present in the room and ensured participants followed these instructions.

Each trial started with a 500 ms presentation of a fixation cross located at the level of the stimulus face's eyes (in the Gaze condition) or bar (in the Arrow condition). Then, a face with a direct gaze (or the bar) appeared on the screen. After 900 ms, the face was replaced by the same face gazing to the right (in half of the trials) or to the left. Thus, in the Gaze condition, participants viewed a face in which the eyes moved away from him/her. In the Arrow condition, the bar was replaced by the arrow pointing to the right (in half of the trials) or to the left. In the Neutral condition, the fixation cross remained on the screen during the whole trial. However, the cross became red between 500 and 900 ms following its appearance and then turned white again. Therefore, the Neutral condition had the same timing as the Gaze and Arrow conditions (Figure 1). In each trial, 500 ms after the last event, the target object appeared at a 11.8° visual angle on the right (in half of the trials) or on the left. The object was aligned with the face's eye and/or with the bar and always appeared on the side indicated by the cue. In the Neutral condition, the object appeared on the right on half of the trials and on the left on the other half. Once the participant responded or after 3,500 ms, a black screen appeared and remained for 900 ms before the next trial. The experiment began with two practice trials that were not analyzed.

For each participant, each of the six conditions [Field of target appearance (Left vs. Right) × Cue (Gaze vs. Arrow vs. Neutral)] was associated with a pair of objects. Six different condition/object pair combinations were created so that, across combinations, each pair of objects was associated with all six conditions. These combinations were counterbalanced across participants. During the experiment, each object appeared five times, always in the same field (right or left) and in the same cue condition (gaze, arrow or neutral).

### Statistical Analyses

For each participant, we computed the Percentage of Correct Responses (%CR) and the mean reaction times of the correct responses (RTs). RTs inferior to 150 ms or exceeding (per subject and per condition) three standard deviations above the mean were rejected. Three Healthy Old participants had aberrant values and were discarded from all the analyses. Two NSU+ patients (2 and 7) had %CR <50% (below chance) and were discarded from all the analyses. Then, for each type of cue (Gaze and Arrow) and each field (Left and Right), we computed the Gain obtained by the presence of the cue. Gain = [RTs for the Neutral condition – RTs for the gaze or arrow condition]. All these variables (%CR, RTs, Gains) were submitted to two repeated measures analyses of variance (ANOVAs) with Cue (Gaze vs. Arrow vs. Neutral for %CR and RTs; Gaze vs. Arrow for Gains) and Field of target appearance (Left vs. Right) as within-subjects factors. The first ANOVA was always restricted to healthy groups and included Age (Young vs. Older) as between-subject factors. Because sex had no significant effect, we removed this variable from all the analyses. The second ANOVA always focused on patients with USN (USN+) and included both USN− and healthy older participants as control groups. Partial Eta-squared ($\eta_{\text{p}}^{2}$) and 90% confidence intervals (CI) are reported as effect size indexes. *Post-hoc* tests with Bonferroni correction were performed when interactions were observed; Cohen's d and 95% CI was used to determine effect size. In healthy groups, the significance of the Gains was tested with bilateral *t*-tests against 0; Cohen's d and 95% CI was used to determine effect size. The normality of distribution was tested with the Kolmogorov-Smirnov Test. The ANOVAs run on %CR and RTs are presented in Supplementary Material.

## Results

### Focus on Healthy Groups

The ANOVA run on Gains revealed a main effect of Age, *F*_(1, 89)_ = 3.99, *p* = 0.049, η^2^_*p*_ = 0.04, 90% CI [0.00, 0.13]. Cuing effects were greater in Young (mean gain = 82 ± 4 ms) than in Older participants (mean gain = 56 ± 7 ms). We also observed a main effect of Cue, *F*_(1, 89)_ = 6.90; *p* < 0.01, η^2^_*p*_ = 0.07, 90% CI [0.01; 0.17]. Participants showed greater gains following Gaze cuing (mean gain = 81 ± 6) than Arrow cuing (mean gain = 65 ± 5).

As the gains did not depend on Field, we averaged right and left gains and tested their significance and distribution, separately for Gaze and Arrow and for Young and Older participants. All gains significantly differed from 0, all *ps* < 0.001, 0.83 < all ds < 1.83, 0.51 < all 95% CI < 2.35, and their distribution did not differ from the normal curve, 0.07 < all ds < 0.10, all *ps* ≥ 0.2.

### Focus on Patients With USN and Their Control Groups

The ANOVA run on Gains showed that Group failed to reach significance, *F*_(2, 58)_ 2.90, *p* = 0.06. However, the interaction between Group and Field was significant, *F*_(2, 58)_ =6.153, *p* < 0.004, η^2^*p* = 0.17, 90% CI [0.04, 0.30]. As expected, in the left field only, cuing effects were greater in USN+ group (mean gain = 183 ± 34 ms) than in Healthy Old [mean gain = 70 ± 14 ms, *p* < 0.001, *d* = 0.73, 95% CI (0.14, 1.56)] and USN– groups [mean gain = 38 ± 31 ms, *p* < 0.001, *d* = 0.91, 95% CI (0.29, 1.81)], all *ps* > 0.1 in the right.

## Discussion

In order to develop a tool that helps neuropsychologist to identify patients with USN who use others' gaze and/or arrows to explore their neglect field, we put into the test, in several populations, a short version of the standard Posner-like paradigm designed to measure gaze and arrow cuing effects. First, our results demonstrated that our test measures very robust cuing effects. They are observed in all populations that we investigated, independent of age, sex or the presence of right brain damage. Importantly, these effects (or gains) followed a normal distribution in healthy populations. Indeed, in neuropsychology, the most common method used to diagnose an individual's behavior and/or cognitive capabilities is to compare his/her performance to a matched control sample (Amieva et al., 2011). Individuals' performance is converted to a z score based on the control group's mean and SD and this z value is referred to a table of areas under the normal curve. In Narison et al. (2019), we proposed using this approach to determine whether a given patient with USN responds to gaze and/or arrow cuing. This is possible if control group performance follows a normal distribution. Our test fits this first condition.

Secondly, comparing individual performance to a control group is possible if the test is adapted to the target population that it aims to test. Our test also fits this second condition. It is noteworthy that patients with USN expressed fewer complaints to the experimenter than in our previous study, in which we manipulated twice as many trials per condition and included an incongruent condition (Narison et al., 2019). Moreover, as expected, patients with USN performed worse (in terms of both %CR and TRs—see Supplementary Material) than healthy older participants, especially in the left field, in accordance with their diagnosed neglect. However, importantly, they showed cuing effects in both fields. As reported in Narison et al. (2019), in their neglect field, these effects were even greater than in healthy older participants. This corroborates our previous conclusion that most patients with USN spontaneously used others' gaze and/or arrows to compensate for their spatial attention deficit (Narison et al., 2019). This corroborates our previous conclusion that most patients with USN spontaneously used others' gaze and/or arrows to compensate for their spatial attention deficit (Narison et al., 2019).

**Figure 2:**
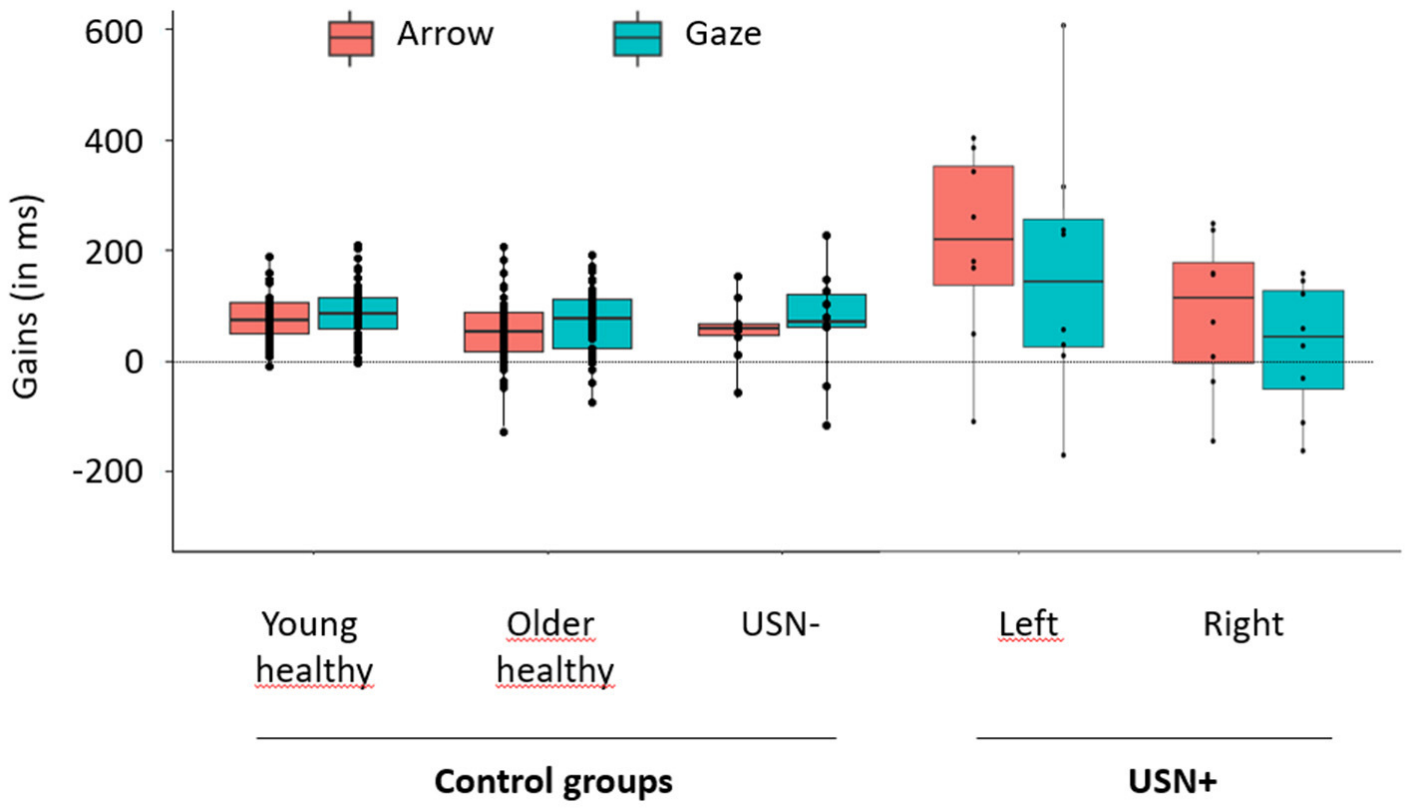
Mean gains obtained in each group for gaze and arrow cues. Arrow conditions are depicted in orange and Gaze conditions in blue. Gains of control groups (young healthy, older healthy, USN–) are depicted on the left part of the graph. As no effect of field was expected or reported in these groups, gains were averaged over the right and left fields. Gains of patients with USN (USN+) are depicted on the right part of the graph. As we expected and reported an effect of Field in this group, gains were represented separately for the right and left fields. The points depicted on each box plot represent the participants' individual mean gain obtained in each condition. Each box plot shows the lower (Q2) and upper (Q3) quartiles, and the horizontal bar inside the box represents the median value of Gain. Vertical bars outside the box represent the distribution range, with the upper bound corresponding to the maximal individual gain and the lower bound corresponding to the lowest individual gain.

This conclusion was further corroborated here by the USN− group that also showed robust cuing effects and did not differ behaviorally from healthy older participants, neither in terms gains, nor in terms of %CR and RTs (see Supplementary Material). The USN+ group displayed particular difficulty on the task, performing worse than the USN– group, both in terms of %CR and RTs (see Supplementary Material). USN+ group show also greater gains than USN– group in the left, converging with the view that cuing effects were intensified by the neglect. It is noteworthy that the cuing effects observed in the USN+ group also showed large standard deviations (see Figure 2), revealing the heterogeneity of the effects, and corroborating the view that patients with USN who do not respond to gaze and/or arrow cues should be distinguished from those who do.

In healthy participants, we observed that young individuals showed greater cuing effects than older individuals, independently of the type of cue (gaze or arrow). This contradicts the idea of a specific age-related decline for gaze cuing compared with arrow cuing (e.g., Slessor et al., 2008, Bailey et al., 2014). This could be explained by number of differences between previous experiments and ours (e.g., the use of incongruent trials, the time of target persistence, the mean age of old participants, the number of trials, ect…). However, our results are in line with Deroche et al. (2016), who showed that age-related differences in cuing effects are linked to general cognitive slowing. Those authors found that gaze cuing culminates for a CTOA of 300 ms in young participants and for a CTOA of 600 ms in older participants. The CTOA of 500 ms we used in the present study seems adequate to measure robust cuing effects in both populations, despite a reduced effect observed among older participants likely related to slowing in executive function.

In healthy participants, we observed that the gaze cuing effect was greater than the arrow cuing effect. This suggests that gaze has a higher alerting value that can be related to its high informative value from the earliest age of human cognitive development (Csibra and Gergely, 2009). However, we did not design the test to study differences between gaze and arrow cuing. The difference we observed could be inherent to the stimuli we included.

## Conclusion

We demonstrated that gaze and arrow cuing effects are powerful enough to be detected in a very short test adapted to the capacities of older patients with severe attention deficits. This emphasizes their applicability in rehabilitation settings. We further argue that the present test fits the criteria that allows us to use it as a basis to develop a tool that will help neuropsychologist to identify patients with USN who use others' gaze and/or arrows to explore their neglect field and who might benefit from this skill as a form of compensation during rehabilitation. The results further show that such a tool should be calibrated in different age groups, as the effects it measures decline with age.

## Data Availability Statement

The datasets presented in this study can be found in online repositories. The names of the repository/repositories and accession number(s) can be found at: https://osf.io/va8c6.

## Ethics Statement

The studies involving human participants were reviewed and approved by CPP Est I, approval no. 2016-A01433-48. The patients/participants provided their written informed consent to participate in this study.

## Author Contributions

LC, MM, and AB designed the tests. RN recorded the participants and analyzed the data. LC and RN wrote the manuscript. All authors contributed to the article and approved the submitted version.

## Conflict of Interest

The authors declare that the research was conducted in the absence of any commercial or financial relationships that could be construed as a potential conflict of interest.
