# Supplementary material for: Measuring Gaze and Arrow Cuing Effects With a Short Test Adapted to Brain Damaged Patients With Unilateral Spatial Neglect: A Preliminary Study
Source: Front Psychol. 2021 Jul 29;12:690197. doi: 10.3389/fpsyg.2021.690197 (PMC8358108; doi:10.3389/fpsyg.2021.690197)
Supplement: Supplementary file 1 [file Data_Sheet_1.docx]

Supplementary Material

# Supplementary results of the main test

Here, we present the result of the ANOVAs run on %CR and RTs obtained in the main test. Before that, we also tested the reliability of our test in all our participants. We estimated the internal consistency of RTs for each conditions using a permutation-based split-half approach (Pronk et al., 2020; Parsons et al., 2019) with 20000 random splits. The Spearman-Brown adjusted Pearson correlation were all comprised between .90 and .95, values that are consensually viewed as very good indices of reliability (Parsons et al., 2019).

## Focus on Healthy Groups

Healthy participants performed very well in all conditions (mean %CR = 99.5 ± 0.1 for Young participants, mean %CR = 99.8 ± 0.1 for Older participants) including catch trials (mean %CR= 99.6 ± 0.1 for Young participants, mean %CR = 99.5 ± 0.0 for Older participants). %CR showed not enough variance to be submitted to an ANOVA (see Table S1).

The ANOVA run on RTs showed a main effect of Age, *F*(1,89 = 48.42, *p* <.001,$\eta_{p}^{2}=.35, 90\% CI [.22, 46]$. Young participants were faster (mean RTs = 399± 16 ms) than Older participants (mean RTs = 557 ± 16 ms). We also observed a main effect of Cue, *F*(2,178) = 36.46, *p* <.001, $\eta_{p}^{2}=.29, 90\% CI [.20,.376]$. Gaze yielded faster RTs than Arrow, *p*=.04 (mean difference = 19 ± 7 ms), which yielded faster RTs than Neutral, p<.001 (mean difference = 64 ± 6 ms). We also observed a main effect of Field, *F*(1,89)=1.32, *p*<.025, $\eta_{p}^{2}=.02, 90\% CI [.00 , .08]$. Participants were faster at detecting the target when it appeared on the right (mean RTs = 471 ± 11 ms) than the left (mean RTs = 485 ± 12 ms). No interaction between factors were observed, all *Fs*<2.2, all *ps*>.1 (see Table S2).

## Focus on patients with USN and their control groups

The ANOVA run on %CR showed a main effect of Group, *F*(2,58) = 19.25, *p* < .01, $\eta_{p}^{2}=.40, 90\% CI [.18,.42]$. USN+ performed worse (mean %CR = 90.8 ± 2.1) than Older participants (mean %CR = 99.8 ± 0.1) and USN- patients (mean %CR = 97.8 ± 0.6). Older participants and USN- patients did not differ on %CR, *p*=1.00. The ANOVA also revealed a main effect of Field, *F*(1,58) = 19.47, *p* < .01, $\eta_{p}^{2}=.25, CI \left[ .10,.39 \right],$ that was modulated by Group, *F*(2,58) = 13.01, *p* < .001, $\eta_{p}^{2}=.31, 90\% CI [.14,.43]$. The effect of Field was significant among USN+ patients only, who performed worse on the left than on the right field, *p*<.03, d=.83, 95% CI [0.37,1.73] (mean difference = 8.3 ± 4.0). No effect of Cue and no interaction were observed, all *Fs*<1, all *ps*>.1 (see Table S1).

The ANOVA run on RTs showed a main effect of Group, *F*(2,58) = 29.20, *p* < .001, $\eta_{p}^{2}=.50, 90\% CI \left[ .33 .60 \right]$. USN+ patients were slower (mean RTs =1212 ± 148 ms) than Older participants, *p* < .001, d=1.98, 95% CI=[1.32,4.04], (mean RTs =557 ± 22ms) and USN- patients, *p*<.001, d=1.78, 95% CI=[1.06, 3.61] (mean RTs =607 ± 53 ms), but Older participants and USN- patients did not differ from each other, *p* = 1.00. Moreover, the ANOVA showed a main effect of Cue, *F*(2,116) = 30.13, *p* < .001, $\eta_{p}^{2}=.34, 90\% CI [.22,.43]$. Neutral condition (mean RTs= 709± 42 ms), yielded faster RTs than Gaze, *p* < .01, d=0.99, 95% CI = [0.74,1.38] (mean RTs= 633± 40 ms) and Arrow, *p* < .01, d=.87, 95% CI = [.62, 1.17] (mean RTs= 639± 37 ms). Interestingly, we observed an interaction between Group and Cue, *F*(4,116) = 3.46, *p* < .02, $\eta_{p}^{2}=0.11, 90\% CI [.01, .17]$, suggesting that cuing effects were modulated by group, as confirmed by the ANOVA run on Gains (see main text). The ANOVA also revealed a main effect of Field, *F*(1,58) = 38.56, *p=* 0.001, $\eta_{p}^{2}=.40, 90\% CI [.23 , .52]$, an interaction between Field and Group, F(2,58)=30.01, p<.001, $\eta_{p}^{2}=.50, 90\% CI [.34 , .61]$, as well as an interaction between Field, Cue and Group, F(4,116)= 2.79; *p* = .03 , $\eta_{p}^{2}=.09, 90\% CI [.00 ,.15]$, suggesting that cuing effects depended on Group and Field as confirmed by the ANOVA run on Gains (see main text).

We further tested whether gains of USN+ group in the neglect field were predicted by some patient’s characteristics. We fitted standard general linear models (GLM) to Gains of Gaze and Arrow separately in the left field. Each of these 2 GLMs included three regressors of interest: the presence/absence of hemaniopia as a categorial predictor, and the post-brain injury delay (in months) and the degree of deviation measured with the Line Bisection test (in millimeters) as continuous predictors. These regression analyses revealed that neither Gaze Gains nor Arrow Gains of USN+ group in the left field were predicted by the post-brain injury delay, by the presence/absence of hemaniopia or by de degree of visual deviation, all *ps* > .1.

## Discussion

As expected, older healthy participants were slower than young healthy participants to perform the task. The USN+ group also displayed particular difficulty on the task, performing worse than the control groups (USN- and older healthy groups), both in terms of %CR and RTs, an effect which is particularly marked in the left field.

Surprisingly, healthy participants were faster at detecting the object when it appeared on the right versus the left, although this effect tended to be limited to Arrow and Neutral conditions. This contradicts the view that mechanisms of spatial attention are usually biased toward the left field (e.g. Zago et al., 2017). We believe that this was driven here by our choice to present all objects’ handles oriented toward the left (see methods). Indeed, handles were more salient when objects appeared on the right than on the left, because they were closer to participants’ central vision. Thus, objects appearing on the right may have favored hand grasp affordances, which are known to activate the motor network and to reduce reaction times (e.g. Tucker & Ellis, 1998, McNair et al., 2017). However, importantly, this effect it did not impact cuing effects that were of similar magnitude in the right and left in all control populations (see main text).

In Narison et al. (2019), we stated that the task should be performed above chance to draw conclusion about the profile of a given patient. In this respect, two patients (no. 2 and no. 7) showed very poor performances (see main text). The exclusion of 20% of patients for poor task performance is consistent with our prior work (see Narison et al., 2019, where 2 of 13 patients were excluded for this reason). These exclusions are themselves informative about severity of cognitive impairment of such patients.

Regression analyses did not reveal any relation between cuing effects (i.e. gains) and the post-brain injury delay, the presence/absence of hemaniopia, or degree of visual deviation. This may be related to the low number of participants and/or heterogeneity in the post-brain injury delay in our sample of patients. Here, we evaluated the reliability of our test in several populations and its feasibility for older patients with USN. Future investigations are needed to determine the profile of patients who respond to gaze and/or arrow cuing.

# Supplementary short tests investigating the gaze liking effect

## Introduction

Because others’ gaze provides information about the environment very early during human development (Csibra & Gergely, 2009), gaze cuing would be special among cuing effects (e.g. Lockhofen, et al. 2014). In line with this view, beyond shifting attention, gaze cuing seems influence preference for the target objects (Van der Weiden et al., 2010). In the Posner-like Paradigm, when gaze is used as the central cue, people tend to prefer target objects processed in congruent trials versus incongruent ones. This is usually not reported when manipulating arrows (e.g. Bayliss et al., 2006, Ulloa et al, 2014, but see Mitsuda et al, 2019 for contradictory results). Interestingly, increasing the saliency and meaningfulness of a stimulus can facilitate its conscious perception by increasing its weight in the competition for attention. Such an effect has been observed in both healthy subjects and patients with spatial neglect (see Vuilleumier, 2005, for a review Dominguez-Borras et al., 2012). The so-called *gaze liking effect* affords an additional way of examining social gaze-facilitated processing in the neglected field.

However, the gaze liking effect has typically been observed when participants completed several blocks of the Posner-like Paradigm (more than 300 trials). The experiment is particularly difficult to administer. Moreover, Tipples & Pecchinenda (2019) recently argued that the gaze liking effect is very small and difficult to detect. It is not clear whether the effect may be observed using a lighter protocol, which questions its applicability for translational research in patient populations. Here, we addressed this issue by adding two additional short tests to our protocol. The first test reproduced what was classically done in the literature but with a limited number of trials, participants completed supplementary Posner-like trials in which they rated the likeability of each target object at the end. The second test was a very simple forced choice procedure, participants were confronted with a pair of objects and had to choose the object they preferred.

In an exploratory way, we thus administrated these two supplementary short tests in all our participants: young adult, older adults, patients with left USN following a right brain lesion (USN+), and patients with right brain lesions but no USN (USN-). Doing this, we tested for the first time the gaze liking effect in patients with right brain damage. We hypothesized that patients with USN would show less preference for objects appearing in their neglect side (vs. the other side) and that such a bias could be attenuated by gaze cuing.

- 1. **Method**

Participants and stimuli were the same than in the main test (see main text).

### Supplementary test 1: testing gaze liking effect with the Posner-like paradigm

***Procedure.*** Supplementary test 1 was inspired by Bayliss et al (2006). The design was the same as in the main test but with only 24 trials, four in each of the six experimental conditions (2 Fields x 3 Cues). No Catch trials were used in Supplementary test 1 (mean test duration: 3 minutes). The condition/object pair combination was the same as in the main test. During the sequence, each object appeared twice, always in the same field (right or left) and in the same cue condition (gaze, arrow or neutral). The structure of the trial was similar to the main test, except that participants had to rate the likeability of the objects. Thus, at the end of each trial, the question “how much did you like that object?” appeared at the top of the screen with a central vertical scale ranging from 1 (“Didn’t like it at all”) to 9 (“Liked it very much”). Participants used the mouse to manipulate the cursor of the scale to provide the response. Once they responded, a black screen appeared for 900 ms, followed by the next trial. The experimenter could help the patient if he/she experienced difficulty using the scale.

***Analyses.*** In healthy groups, Correct Responses (%CR), the mean Reaction times of the correct responses (RTs), the Gains and the mean object ratings were each submitted to a repeated measure analyses of variance (ANOVA) with Cue (Gaze vs. Arrow vs. Neutral for %CR and RTs ; Gaze vs. Arrow for Gains) and Field of target appearance (Left vs. Right) as within-subjects factors and Age (Young vs Older) as between subject factors. Partial Eta-squared (η^2^_p_) and 90% confidence intervals (CI) are reported as effect size indexes. Post-hoc tests with Bonferroni correction were performed when interactions were observed; Cohen’s d and 95% CI was used to determine effect size. Since supplementary test 1 tested cuing effects using very few trials (4 per conditions), and that USN+ patients made several errors, we did not analyze %CR, RTs and Gains of this group. The analysis focusing on USN+ patients was restricted to the mean object ratings that was submitted to a repeated measure analyses of variance (ANOVA) with Cue (Gaze vs. Arrow vs. Neutral) and Field of target appearance (Left vs. Right) as within-subjects factors and Group (Older, USN-, USN+) as between subject factors. Partial Eta-squared (η^2^_p_) and 90% confidence intervals (CI) are reported as effect size indexes. Planned comparisons were performed when interactions were observed; Cohen’s d and 95% CI was used to determine effect size.

### Supplementary test 2: testing gaze liking effect with a forced choice procedure

***Procedure.*** Eighteen trials consisted of a forced choice procedure between new pairs of objects. At each trial, two objects appeared on the screen, one above a fixation cross and the other one below it. Participants had to select their preferred object using the mouse. The experimenter could help the patient if he/she had difficulty using the mouse. The objects remained on the screen until the participant’s response (mean test duration: 1 minute).

Among the 18 pairs of objects presented during the sequence, nine pairs presented two large objects and nine pairs presented two small objects. Also, six pairs presented objects that always appeared on the left during the two previous tests (Left-Objects) while another six pairs presented Right-Objects. Within these two sets of six pairs, two pairs presented an object in the Gaze condition (a Gaze-Object) with an Arrow-Object, two other pairs presented a Gaze-Object with a Neutral-Object, and the last two pairs presented an Arrow-Object with a Neutral-Object. The last six pairs presented a Right-Object with a Left-Object presented in the same Cue condition (2 pairs with Gaze-Objects, 2 pairs with Arrow-Objects, and 2 pairs with Neutral-Objects).

The 18 pairs of objects were the same for all participants, but the order of pair presentation was randomly presented to each participant. Moreover, at each trial, the program randomly determined which of the two objects appeared above or below the fixation cross (which was located at the same place in the main test and the supplementary test 1).

***Analyses.*** For each participant, we computed the number of times objects in each condition were chosen, separately for each group of healthy participants (Older vs. Young) and patients (USN- vs. USN+). This variable did not follow a normal distribution (d=0.22, p<.05 at the Kolmogorov-Smirnov Test). We thus applied a non-parametric Friedman’s ANOVA with Cue (Gaze, Arrow, Neutral) as a within-subject factor. A Wilcoxon test was also applied to compare between Fields (Left vs. Right).

## Results

### Supplementary test 1: testing gaze liking effect with the Posner-like paradigm

### Focus on Healthy Groups. Healthy participants performed very well in all conditions (mean %CR = 99.2 ± 0.3 for Young participants, mean %CR = 98.1±0.7 for Older participants).

The ANOVA run on RTs showed a main effect of Age, *F*(1,89) = 46.98, *p* < .001,$\eta_{p}^{2}=.35, 90\% CI [.22,.46]$. Young participants were faster (mean RTs =452 ± 51 ms) than Older participants (mean RTs = 954 ± 53 ms). We also observed a main effect of Cue, *F*(2,178) = 39.86, *p* < .001,$\eta_{p}^{2}=.30, 90\% CI [.21 , .38]$. Participants were slower in the Neutral condition (mean = 766 ± 45 ms ) than in the Arrow (mean = 662 ± 47, *p* < .001, d = .82, 95% [0.65,1.19]) and Gaze conditions (mean = 642 ± 45, *p* < .001, d = .83, 95% [0.61,1.14]). No difference was observed between Gaze and Arrow. Finally, no other effects or interactions were observed, all *Fs*<1, all *ps*>.1.

The ANOVA run on Gains showed no main effect of Age, Cue or Field, nor interactions between these factors, all *Fs*<1, all *ps*>.1. All *Fs*<1, all *ps*>.1.

The ANOVA run on mean object ratings showed no main effect of Age, Cue or Field, nor interactions between these factors, all *Fs*<1, all *ps*>.1 (see Table S3).

**Focus on patients with USN and their control groups.** The ANOVA run on object ratings showed a main effect of Field, *F*(1,58) = 5.31, *p* = .02, $\eta_{p}^{2}=.09, 90\% CI [.01, .21]$. Participants preferred objects that appeared on the right than on the left. Importantly, however, this effect was modulated by Groups, *F*(2,58) = 3.82, *p* < .03, $\eta_{p}^{2}=.12 , 90\% CI [.01,.23]$. As expected, planned comparisons revealed that the effect of Field reached significance in the USN+ group, *p* *<.*05, $d=.78, 95\% CI \left[ .26, 1.63 \right]$ (mean rating on the right = 5.6 ± 0.4, mean rating on the left = 4.8 ± 0.4), but not in the USN- group, *p*=.23, or among Older participants, *p*=.65. No other effects or interactions were observed, all *Fs*<2.0, all *ps*>.1.

**Supplementary test 2: testing gaze liking effect with a forced choice procedure**

A Friedman’s ANOVA run on mean chosen objects per Cue Condition showed no effect of Cue in Young participants, *χ^2^* = 0.47, *p* >.1, Older participants, *χ^2^* = 5.12, *p* = .08, USN+ ,χ*^2^* = 4.08, *p* > .1, or USN-, χ*^2^* = 1.88, *p* >.1. The Wilcoxon Tests run on mean chosen objects per Field showed no significant difference between left and right in Young participants, *p*=0.64, Older participants, *p* = 0.72, USN+ , *p* = 0.68 or USN- *p* = 0.36 (see Table S4).

## Discussion

In two independent supplementary short tests, we failed to report gaze liking effect. This effect is obviously much more discrete than the cuing effect and may thus have been difficult to reach with such a limited number of trials (see Tipples and Pecchinenda, 2019 for a similar proposal). We conclude that the gaze liking effect is not robust enough to be measured with a light protocol, which limits its interest for translational research in patient populations.

As expected, analyses run on supplementary test 1 showed that patients with USN preferred objects that appeared on the right relative to the left in the Posner-like task. By contrast to our hypothesis however, gaze did not attenuate this effect. Moreover, the absence of effect observed on the forced choice procedure developed for supplementary test 2 suggests either that the effect of field on object’s preference observed in the Posner-Like task is short live, or that this procedure is not suitable for investigating liking effect related to the Posner-Like task.

Since supplementary test 1 tested cuing effects using very few trials (4 per conditions), the results have to be taken with caution. Still, analyses run on RTs highlighted that, even in such condition, gaze and arrow cuing effects are observed in healthy populations (Young healthy, Older healthy), converging with the view that these effects are highly robust and reproducible.

# Supplementary references

Tucker, M., & Ellis, R., (1998). On the relations between seen objects and components of potential actions. *Journal of Experimental Psychology: Human Perception and Performance*, 24,830–846.

McNair, N.A., Behrens, A.D. & Harris, I. (2017). Automatic recruitment of the motor system by undetected graspable objects : A motor-evoked potential study. *Journal of Cognitive Neuroscience*, 29, 11, 1918-1931.

Tipples, J. & Pecchinenda, A. (2019). A closer look at the size of the gaze-liking effect : a preregistered replication. *Cognition and Emotion,* 33(3), 623-629.

Zago, L., Petit, L, Jobard, G., Hay, J., Mazoyer, B., Tzourio-Mazoyer, N., Karnath, HO. & Mellet, E. (2017). Pseudoneglect in line bisection judgement is associated with a modulation of right hemispheric spatial attention dominance in right-handers. *Neuropsychologia*, 94, 75-83.

Parsons, S., Kruijt, A.-W., and Fox, E. (2019). Psychological science needs a standard practice of reporting the reliability of cognitive-behavioral measurements. *Adv. Methods Pract. Psychol. Sci.* 2, 378–395. [doi: 10.1177/2515245919879695](https://doi.org/10.1177/2515245919879695)
sss
Pronk, T., Molenaar, D., Wiers, R., and Murre, J. (2020). *Methods to Split Cognitive Task Data for Estimating Split-Half Reliability: A Comprehensive Review and Systematic Assessment*. [doi: 10.31234/osf.io/ywste](https://doi.org/10.31234/osf.io/ywste)

# Supplementary Tables

| **% Correct responses** | | **Left Field** | **Right Field** |
| --- | --- | --- | --- |
| **Young participants** | **Gaze** | 99.8±0.0 | 100±0.0 |
|  | **Arrow** | 99.8±0.0 | 99.8±0.0 |
|  | **Neutral** | 99.1±0.0 | 98.9±0.0 |
| **Older participants** | **Gaze** | 100±0.0 | 99.7±0.2 |
|  | **Arrow** | 99.8±0.2 | 99.8±0.2 |
|  | **Neutral** | 99.5±0.3 | 99.8±0.2 |
| **USN-** | **Gaze** | 97.0±1.5 | 99.0±1.0 |
|  | **Arrow** | 97.0±1.5 | 97.0±1.5 |
|  | **Neutral** | 98.0±2.0 | 99.0±1.0 |
| **USN+** | **Gaze** | 85.0±5.9 | 92.5±3.6 |
|  | **Arrow** | 90.0±4.2 | 96.2±1.8 |
|  | **Neutral** | 85.0±8.8 | 96.2±2.6 |

**Table S1.** Mean percentage of correct responses (%CR) with standard error (±) for the Young and Older healthy groups (upper part), and for the patients with and without USN (USN+ and USN-, bottom part) in each condition of the main test. Healthy participants and USN- patients performed at ceiling in all conditions. USN+ patients performed worse than the control groups (older participants and USN-) but better in the right than the left field.

| **Reaction times (ms)** | | **Left Field** | **Right Field** |
| --- | --- | --- | --- |
| **Young participants** | **Gaze** | 367±11 | 363±10 |
|  | **Arrow** | 386±11 | 368±10 |
|  | **Neutral** | 461±12 | 447±9 |
| **Older participants** | **Gaze** | 533±29 | 540±32 |
|  | **Arrow** | 563±29 | 537±21 |
|  | **Neutral** | 623±29 | 585±20 |
| **USN-** | **Gaze** | 574±47 | 575±48 |
|  | **Arrow** | 595±55 | 588±64 |
|  | **Neutral** | 626±56 | 670±59 |
| **USN+** | **Gaze** | 1257±150 | 961±124 |
|  | **Arrow** | 1211±163 | 900±106 |
|  | **Neutral** | 1418±193 | 984±119 |

**Table S2.** Mean reaction times (RTs in ms) with standard error (±) for Young and Older healthy groups (upper part), and for patients with right brain damage with and without USN (USN+ and USN-, bottom part) in all experimental conditions of the main test. USN+ patients were slower than other groups, and they were slower in the left relative to the right field.

| **Ratings** | | **Left Field** | **Right Field** |
| --- | --- | --- | --- |
| **Young participants** | **Gaze** | 5.2±0.2 | 5.0±0.2 |
|  | **Arrow** | 5.3±0.2 | 5.1±0.2 |
|  | **Neutral** | 5.4±0.2 | 5.0±0.2 |
| **Older Participants** | **Gaze** | 5.3±0.2 | 5.3±0.2 |
|  | **Arrow** | 5.4±0.2 | 5.1±0.2 |
|  | **Neutral** | 5.4±0.2 | 5.4±0.2 |
| **USN-** | **Gaze** | 4.5±0.5 | 4.8±0.5 |
|  | **Arrow** | 4.5±0.5 | 4.7±0.3 |
|  | **Neutral** | 4.4±0.3 | 5.1±0.6 |
| **USN+** | **Gaze** | 5.1±0.5 | 5.5±0.6 |
|  | **Arrow** | 4.0±0.5 | 5.6±0.5 |
|  | **Neutral** | 5.2±0.3 | 5.8±0.5 |

**Table S3.** Mean ratings with standard error (±) for Young and Older healthy groups (upper part), and for patients with right brain damage with and without USN (USN+ and USN-, bottom part) in all experimental conditions of part 2. USN+ patients rated objects that appeared on the right slightly higher than objects that appeared on the left neglect side.

| **Mean number of chosen objects** | | |
| --- | --- | --- |
| **Young participants** | **Gaze** | 3.8±0.2 |
|  | **Arrow** | 4.0±0.2 |
|  | **Neutral** | 4.2±0.2 |
|  | **Right** | 2.9±0.2 |
|  | **Left** | 3.1±0.2 |
| **Older Participants** | **Gaze** | 4.2±0.2 |
|  | **Arrow** | 3.5±0.2 |
|  | **Neutral** | 4.6±0.2 |
|  | **Right** | 3.0±0.2 |
|  | **Left** | 3.2±0.2 |
| **USN-** | **Gaze** | 4.1±0.6 |
|  | **Arrow** | 3.7±0.5 |
|  | **Neutral** | 4.6±0.6 |
|  | **Right** | 2.6±0.5 |
|  | **Left** | 3.4±0.5 |
| **USN+** | **Gaze** | 3.2±0.4 |
|  | **Arrow** | 4.0±0.4 |
|  | **Neutral** | 4.7±0.3 |
|  | **Right** | 3.1±0.5 |
|  | **Left** | 2.8±0.5 |

**Table S4.** Mean number of chosen objects with standard error (±) for Young and Older healthy groups (upper part), and for patients with right brain damage with and without USN (USN+ and USN-, bottom part) in all experimental conditions of part 3.
